# Identification of compositional and structural changes in the nucleus pulposus of patients with cervical disc herniation by Raman spectroscopy

**DOI:** 10.3389/fendo.2022.1015198

**Published:** 2022-10-07

**Authors:** Zhiqi Wang, Tao Xue, Tongxing Zhang, Xuehui Wang, Hui Zhang, Zhongyu Gao, Qiang Zhou, Erke Gao, Tao Zhang, Zhaoyang Li

**Affiliations:** ^1^ Department of Orthopedic, Tianjin First Central Hospital, Tianjin, China; ^2^ School of Materials Science and Engineering, Tianjin University, Tianjin, China; ^3^ Department of Minimally Invasive Spine Surgery, Tianjin Hospital, Tianjin University, Tianjin, China; ^4^ Department of Orthopedic and Joint Sports Medicine, The First Affiliated Hospital of Baotou Medical College, Baotou, China; ^5^ First Central Clinical College, Tianjin Medical University, Tianjin, China

**Keywords:** intervertebral disc degeneration, cervical disc herniation, Raman spectroscopy, nucleus pulposus, proteoglycan

## Abstract

**Purpose:**

Cervical disc herniation (CDH) is one of the most common spinal diseases in modern society; intervertebral disc degeneration (IVDD) has long been considered as its primary cause. However, the mechanism of intervertebral disc degeneration is still unclear. The aim of the study is to examine the components and structures of proteoglycan and collagen in cervical disc herniated nucleus pulposus (NP) using a validated and convenient Raman spectra technique and histological methods to further elucidate the mechanism of IVDD at the microscopic level.

**Methods:**

Our study used a burgeoning technique of Raman spectroscopy combined with *in vitro* intervertebral disc NP to characterize the above mentioned research purposes. Firstly, we collected cervical disc NP samples and imaging data by certain inclusion and exclusion criteria. Then, we graded the NP of the responsible segment according to the patient’s preoperative cervical magnetic resonance imaging (MRI) T2-weighted images by Pfirrmann grading criteria while measuring the T2 signal intensity value of NP. In addition, the structure of the NP samples was evaluated by histological staining (H&E staining and Safranin-O staining). Finally, the samples were scanned and analyzed by Raman spectroscopy.

**Results:**

A total of 28 NP tissues from 26 patients (two of these patients were cases that involved two segments) with CDH were included in this study. According to the Raman spectroscopy scan, the relative content of proteoglycans which is characterized by the ratio of the two peaks (*I*_1,064/_ *I*_1,004_) in the NP showed a significantly negative correlation with Pfirrmann grade (*P* < 0.001), while the collagen content and the NP intensity value showed a positive correlation (*P* < 0.001). For the microstructural characterization of collagen, we found that it may have an essential role in the degenerative process of the intervertebral disc. Moreover, histological staining (H&E staining and Safranin-O staining) showed the general structure of the NP and the distribution of macromolecules.

**Conclusion:**

The present study demonstrated the possibility of characterizing the macromolecular substances inside the cervical disc NP tissue by Raman spectroscopy. It also confirmed that macromolecular substances such as proteoglycans and collagen have some degree of alteration in content and structure during degeneration, which has a further positive significance for the elucidation of CDH’s mechanism.

## Introduction

Cervical disc herniation (CDH), as one of the most prevalent spinal diseases, is characterized by radicular symptoms and spinal cord compression symptoms such as neck and shoulder pain, muscle tone changes, and so on. CDH is considered to be a highly prevalent disease in middle-aged and elderly people (1), but with the popularization of social lifestyles and electronic devices, it is gradually showing a trend of rejuvenation (2). A review of the global burden of neck pain estimated that more than one-third of the world’s population experience persistent neck and shoulder pain malaising for more than 3 months (3), further emphasizing the magnitude of CDH’s impact on global health. The occurrence of degenerative diseases such as CDH and depression-related psychiatric disorders has been well documented (4), causing physical pain as well as mental health risks to patients. As the earliest and most susceptible of the body’s tissues to succumb to degeneration with age, the intervertebral discs also lose water and corresponding elasticity in the NP (5). Intervertebral disc degeneration (IVDD) is also considered to be an influential risk factor for the development of CDH (6). Although there are a fair number of theories and related animal studies in the exploration of IVDD, the internal composition and microstructural changes of the human intervertebral disc NP remain a significant challenge for scientific research.

The high sensitivity of MRI to water and proteoglycan content has made it widely used in the diagnosis of intervertebral disc- and cartilage-related diseases (7). However, MRI is more of an adjunctive diagnostic tool for clinically relevant diseases and is still limited in its ability to characterize the macromolecular substances within the NP during IVDD. In the maturation of MRI-related technologies, there has been no shortage of approaches to improve the accuracy of diagnosis through dynamic magnetic resonance imaging, functional magnetic resonance imaging, diffusion imaging, *etc.* (8). Nevertheless, there are still several restrictions of MRI in evaluating microstructural changes within the NP.

Raman spectroscopy, being a new generation of non-destructive molecular spectroscopy, has been widely used in recent years for the study of structural and compositional changes of biomolecules in diseased tissues and in the early diagnosis of related diseases (9). It has the advantages of real-time rapid detection, simple sample processing, low biological interference, and high sensitivity and specificity (10, 11); most importantly, it can indirectly reflect the structural composition and content changes of macromolecules at the microscopic level (12), which makes it one of the hot spots for the early diagnosis of related diseases (13). It has also been reported in the literature that it is used in the study of articular cartilage and lumbar intervertebral disc degeneration.

We analyzed the NP tissue of cervical discs with different degrees of degeneration by Raman spectroscopy to further investigate the mechanism of disc degeneration and the feasibility of early clinical diagnosis.

## Materials and methods

### Patient data and sample collection

Our study was endorsed by the Ethics Committee of Tianjin First Central Hospital, and the consent of patients with CDH treated by surgery was obtained. All procedures were carried out in strict compliance with relevant domestic and international policies. A total of 28 NP samples from 26 patients (15 male and 11 female; age, 20–78 years; mean age, 51.4 years) with CDH treated by anterior cervical spine surgery in the Department of Orthopedics, Tianjin First Central Hospital, were enrolled in this study from June 2020 to September 2021. All patients included in the group were diagnosed with CDH by MRI and had relevant clinical symptoms and corresponding syndromes, among whom conservative treatment was found ineffective after 3 months of application.

### Preoperative Pfirrmann grade

All patients’ cervical MRI profiles were scanned at the Siemens Magnetom Trio 3.0T superconducting MR scanner (Magnetom, Trio, Siemens Healthcare, Erlangen, Germany) in the imaging department of the First Central Hospital of Tianjin. The cervical MR scan parameters were as follows: sagittal T2-weighted: repetition time, 3,270 ms; echo time, 112 ms; field of view, 260 × 260 mm; slice thickness, 3.0 mm; scanning slice number, 12; and voxel size, 320 × 224 mm. The samples were graded and evaluated on MRI sagittal T2-weighted images by the clinically used Pfirrmann grading (14), an intervertebral disc degeneration classification standard. The images were evaluated by a radiologist and a spine surgeon who were both unaware of the experiment, and the observers graded the sample strictly according to the Pfirrmann grading in the median sagittal position of the cervical MRI T2-weighted term. To increase the accuracy of the grading results, the cervical spine images of all patients were randomly evaluated by both observers again 2 months later (the two observers were unaware of the homogeneity of the two evaluations).

### NP signal intensity value measurement

We used Siemens post-processing software to measure the signal intensity of NP on MRI sagittal T2-weighted images. The study randomly selected a range area (20–40 mm^2^) of anterior, middle, and posterior positions on the targeted NP and then measured three times continuously at the respectively selected position to obtain the average brightness value. The brightness value of the cerebrospinal fluid on the cervical segment was measured by a similar method. Then, the NP signal intensity value was defined as the ratio of NP to the cerebrospinal fluid brightness value (15).

### Preparation of sample

All samples were obtained through anterior cervical surgery in strict accordance with the surgical procedure, then placed in sterile sample boxes, and labeled with patient-related information under the premise of sample freezing. The samples were repeatedly washed with sterile saline in clean petri dishes. The loose NP tissues were blended into ellipsoidal spheres with a radius of 3 mm (for better embedding and section staining) and placed in optimal cutting temperature (OCT) media for embedded freezing treatment. All samples were processed by frozen sectioning to a thickness of 30 μm on stripping-resistant slides, and the samples were collected in a slide box and stored in a refrigerator at -20°C.

### Assessments of histological staining

The obtained frozen section samples were removed from -20​°C inside the refrigerator and thawed to room temperature for histological staining (1). Hematoxylin and eosin (H&E) staining was carried out as follows: the OCT-embedded frozen sections were washed in running water at room temperature for 5 min. Subsequently, the frozen sections were stained with hematoxylin (H&E staining kit; Beijing Solarbio Science and Technology Co., Ltd.) for 10 ​min, followed by fractionation in 1% hydrochloric acid alcohol for 2 s. Then, the sections were incubated in 1% ammonia for 2 min and stained with 1% eosin for 1 min. After each step, the sections were rinsed under running water for 3 min. Lastly, the sections were dehydrated with graded ethanol and vitrified with dimethylbenzene. Then, the sections were sealed with neutral resin. After that, the upper layer of each group of sample tissue was stained, dried, and observed under a microscope, with the position of the corresponding NP tissue being marked. The corresponding position of the lower layer of the sections was marked as well. All processes were performed under sterile conditions (2). On the other hand, Safranin-O-Fast Green staining (Modified Safranine O-Fast Green FCF Cartilage Stain Kit; Beijing Solarbio Science and Technology Co., Ltd.) was performed as follows: OCT-embedded frozen sections were washed in running water at room temperature for 5 min, and then the sections were stained with freshly prepared Weigert iron hematoxylin stain for 5 min before being washed under running water for 3 min. Then, the sections were partitioned in acidic partitioning solution for 15 s and rinsed in distilled water for 10 min. After immersing the sections in Fast Green solution for 5 min, the sections were quickly washed with a weak acid solution for 15 s. The sections were dried before being stained in Safranin-O solution for 3 ​min. Finally, the sections were dehydrated, vitrified, and mounted with neutral gum.

All staining procedures were performed according to the kit manufacturer’s instructions. The images of the stained sections were obtained using an optical microscope (Nikon Eclipse 600, Nikon Corporation) mounted with a digital camera (Nikon DXM1200F, Nikon Corporation).

### Sample Raman spectroscopy detection

All frozen samples were defrosted to room temperature, and the samples were rinsed with sterile saline to wash off the OCT reagent for Raman spectroscopy. Sterile saline was added dropwise to keep the samples moist during the testing process (the characteristic peaks of saline on the Raman spectra were negligible). The Raman spectroscopy test parameters were as follows: laser wavelength, 532 nm; sample magnification, ×500; laser power, 10.0 mW; spot size, 1.1 μm; spectral range, 800–1,800 cm^-1^; grating, 900 line/mm; and resolution, 5 cm^-1^. For each NP sample, a total of 10 randomly selected positions were scanned by the spectrometer to obtain the corresponding spectra (16), with a single exposure time of 16 s and number of scans of 5. Confocal correction was needed before scanning at different positions.

The acquired Raman spectral data were processed and analyzed by OMNIC software (Thermo Fisher Scientific, Inc.), and the peak shapes and peak positions were calculated by fitting the Raman spectra with Gaussian/Lorentzian functions after baseline correction. Then, the intensity and the area of the different peaks of the obtained Raman spectra were imported into Microsoft Excel software to calculate the relative ratios of intensity and area of different characteristic peaks.

### Statistical analysis

Statistical analysis was performed using SPSS 26.0 software (SPSS, Chicago), and all data were expressed as mean ± standard deviation. *κ* statistics was used to evaluate the consistency and reliability of Pfirrmann grade results. The association between the NP T2-weighted intensity values and the relative content of proteoglycans was assessed by a bivariate correlation. Statistical differences between groups were statistically analyzed using one-way ANOVA, followed by Tukey’s test for variability between the Raman spectral data of different Pfirrmann grades. The correlation between the acquired Raman spectral data and Pfirrmann grade was assessed by Spearman’s correlation analysis. *P <*0.05 was defined as statistically significant.

## Results

### Pfirrmann classification

**Figure 1:**
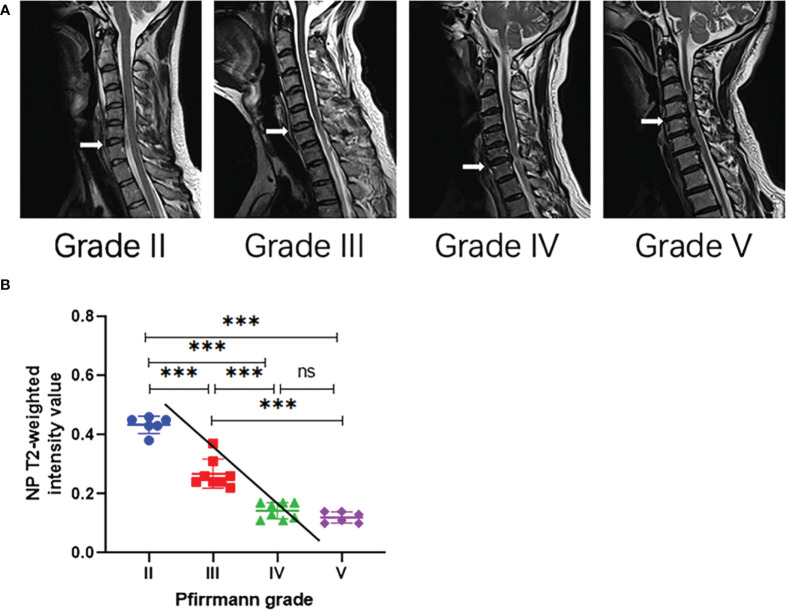
**(A)** Representative T2-weighted median sagittal MRI scans of the cervical spine with intervertebral disc degeneration at different Pfirrmann grades. The segments responsible for the lesion are indicated by the white arrow. **(B)** Analysis of NP T2 signal intensity values with Pfirrmann grade by Spearman’s correlation analysis. ^∗∗∗^ *P* < 0.001; ns, no significance.

By *κ* statistics analysis, the first grading results were in great agreement between the two observers (*κ* = 0.620, *P* < 0.001), and the second observation grading results were in excellent agreement likewise (*κ* = 0.614, *P* < 0.001). The grading results of patients with CDH included in this study did not have Pfirrmann grade I (**Table 1**). The imaging performance of patients with a different grading is shown in **Figure 1A**.

**Table 1:** Grading results of patients with Pfirrmann grade.

Pfirrmanngrade	Sex		Intervertebral disc level				Total
	Male	Female	C3/4	C4/5	C5/6	C6/7
II	2	4	1	1	3	1	6
III	5	1	1	1	3	3	8
IV	5	3	4	1	3	0	8
V	3	3	3	1	2	0	6
Total	15	11	9	4	11	4	28

### Analysis of the NP T2 signal intensity value

The present study demonstrated that the NP T2 signal intensity value in Pfirrmann III, IV, and V was significantly lower compared with Pfirrmann II (*P* < 0.001), but the NP T2 signal intensity value was not significantly different between grade IV and grade V (*P* > 0.05). In terms of Spearman’s correlation analysis, there was a significantly negative correlation between NP T2 signal intensity value and Pfirrmann grade (*ρ* = -0.9196, *P* < 0.001, **Figure 1B**).

### Histological staining of NP in different Pfirrmann grades

#### H&E staining

**Figure 2:**
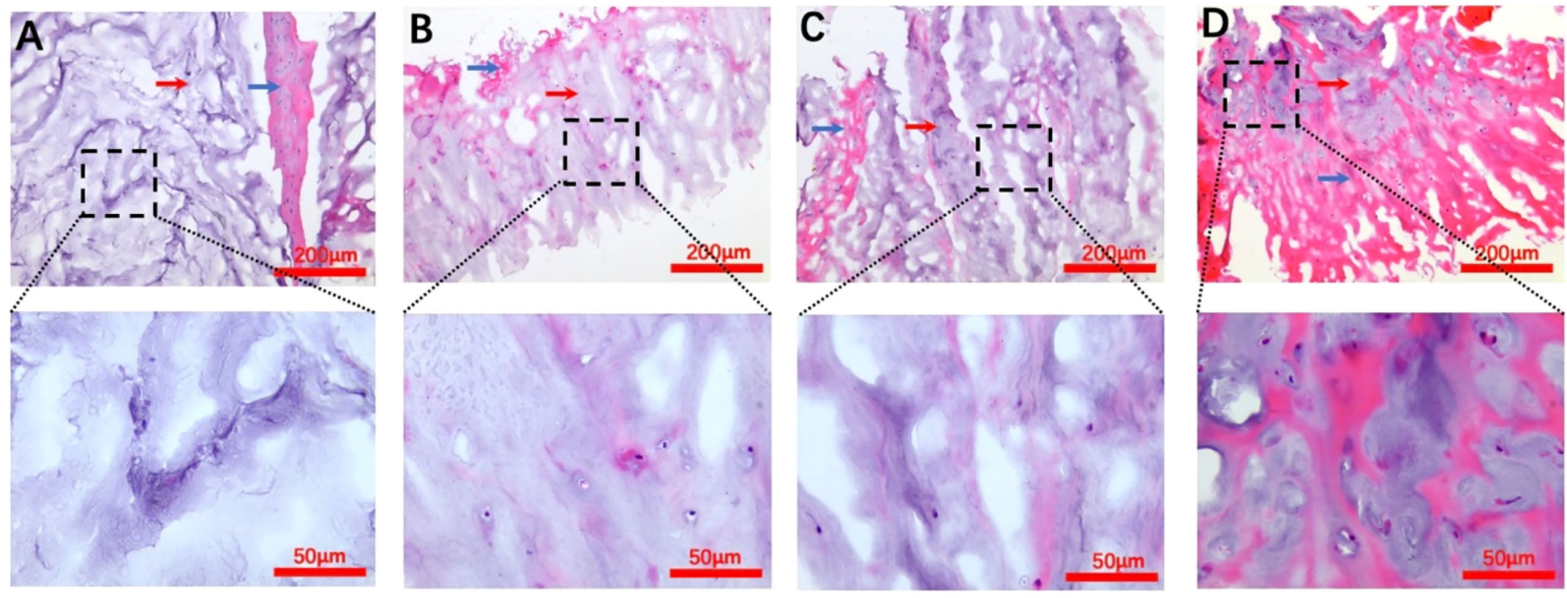
H&E staining images for each Pfirrmann grade. **(A–D)** Representative Pfirrmann grade II, III, IV, and V images, respectively. The blue arrows in each figure point to the annulus fibrosus tissue, and the nucleus pulposus tissue structure is indicated by the red arrows. The scale bar of each figure is shown at the lower righthand corner of the figure.

In the normal macroscopic assessment, the annulus fibrosus (AF) tissue was dense and showed the typical several layers of concentric fibrous sheets, whereas since our study used a surgically obtained sample, it was possible to see the presence of partial AF tissues presenting as red striated structures, but there were more pale blue NP tissues in the field of view, which appeared spongy and had a soft and loose appearance. In addition, scattered nuclei could be seen in the extracellular matrix of the NP (**Figure 2**).

#### Safranin-O-Fast green staining

**Figure 3:**
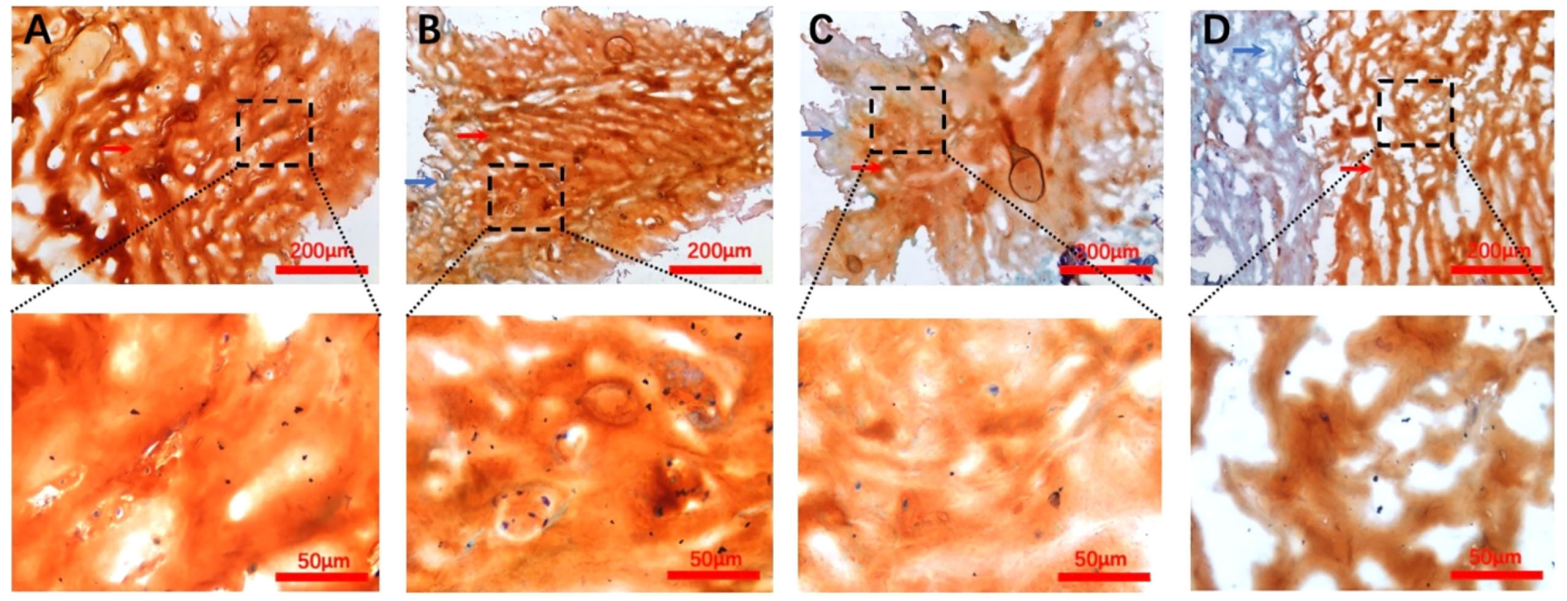
Safranin-O-Fast Green staining images for each Pfirrmann grade. **(A–D)** Representative Pfirrmann grade II, III, IV, and V images, respectively. The blue arrows in each figure point to the annulus fibrosus tissue, and the nucleus pulposus tissue structure is indicated by the red arrows. The scale bar of each figure is shown at the lower righthand corner of the figure.

All grade samples were stained as shown in **Figure 3**; we observed the structural components of the NP proteoglycan to be showing a reddish tone as well as the fibrous ring tissue in pale blue color. The proteoglycan structure was fluffy and more abundantly distributed in the extracellular matrix than the AF tissue. Furthermore, Safranin-O staining was also used to appropriately express the distribution of proteoglycans in the extracellular matrix. Another reason for these procedures was to make sure that all locations tested were NP tissue, and the same position was accurately detected by subsequent Raman spectroscopy.

### Raman spectroscopy peak distribution

Proteoglycans and collagens play a major role in the extracellular matrix of NP (17), and macromolecules like these all have their own characteristic peaks in Raman spectroscopy. Proteoglycans are often characterized in Raman spectroscopy by their special glycosaminoglycan complex structure, which has been previously identified by scholars, and their characteristic peaks are mainly located at 1,064–1,065 cm^-1^ (18); phenylalanine is often used as a reference internal standard to measure the relative content due to its insensitivity to the surrounding environment, and its characteristic peak position on the Raman spectrum is mainly in the range of 1,003–1,004 cm^-1^ (19). Because of the possibility of individual differences in the proteoglycan content within the sample, the measured proteoglycan Raman spectral peak intensities are usually corrected by the ratio for further comparison. Therefore, the ratio of the two (*I*_1,064_/*I*_1,004_) was taken as the relative content of proteoglycan.

Collagen, as an internal mesh-mounted scaffold structure for intervertebral disc, has its peak characterization on Raman spectra mainly depending on two amide groups: amide I and amide III. The identification of the peak positions of the two Raman spectra has been demonstrated in the last century: the peak position of amide I is mainly located at 1,600–1,700 cm^-1^ (20, 21), while amide III is at 1,200–1,300 cm^-1^ (20, 22).

**Figure 4:**
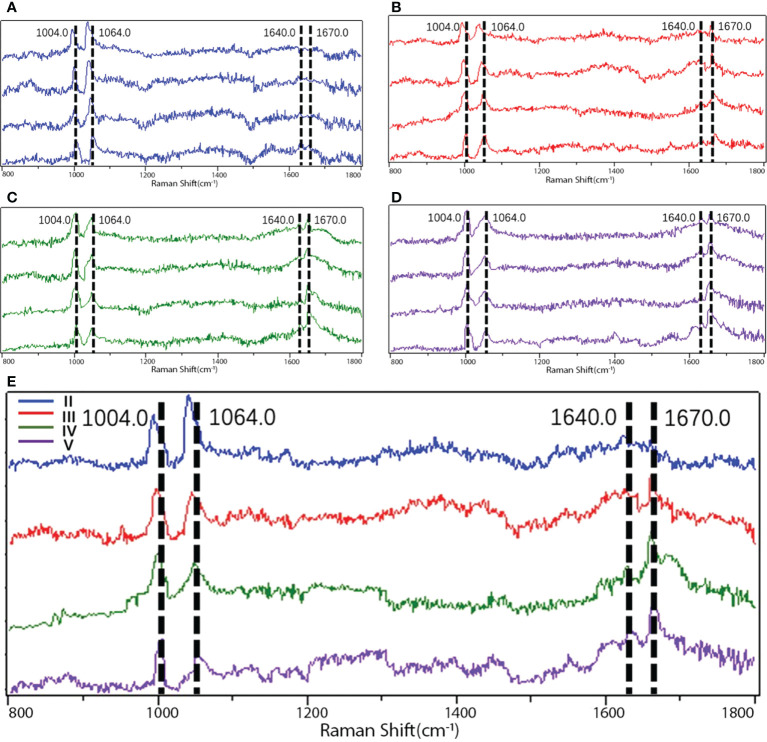
Raman spectra images of different Pfirrmann grades. **(A)** Images detected by Raman spectroscopy in Pfirrmann grade II. **(B)** Images detected by Raman spectroscopy in Pfirrmann grade III. **(C)** Images detected by Raman spectroscopy in Pfirrmann grade IV. **(D)** Images detected by Raman spectroscopy in Pfirrmann grade V. **(E)** Comparison among the images in different Pfirrmann grades detected by Raman spectroscopy. *I*_1,004.0_, peak position of phenylalanine; *I*_1,064.0_, peak position of proteoglycan; *I*_1640.0_, peak position of α-helix in amide I; *I*_1670.0_, peak position of random coil in amide I; *I*, relative intensity.

In contrast, the collagen secondary structure changes are mainly characterized by correlated Raman spectroscopy peaks within both, and the ratio of the correlated peaks is usually used to assess the relative variation in disordered collagen (random coil) *versus* ordered collagen (α-helix) (20, 23, 24). The peak positions of the Raman spectra of various macromolecules in the samples are provided in **Table 2**, and the fitted and calculated images of different Pfriimann-graded Raman spectra are shown in **Figure 4**.

**Table 2:** Raman spectroscopy peak distribution and affiliated compounds.

Peak position(cm^-1^)	Affiliated compounds	References
1,004	Phenylalanine	(19)
1,064	Glycoaminoglycan	(18)
1,200–1,300	Amide III, major collagen band	(20, 22)
1,640	Amide I, ordered coil, α-helix	(20, 23, 24)
1,670	Amide I, disordered coil, random coil	(20, 23, 24)
1,600–1,700	Amide I, major collagen band	(20, 22)

### Analysis of proteoglycan and collagen content in samples

**Figure 5:**
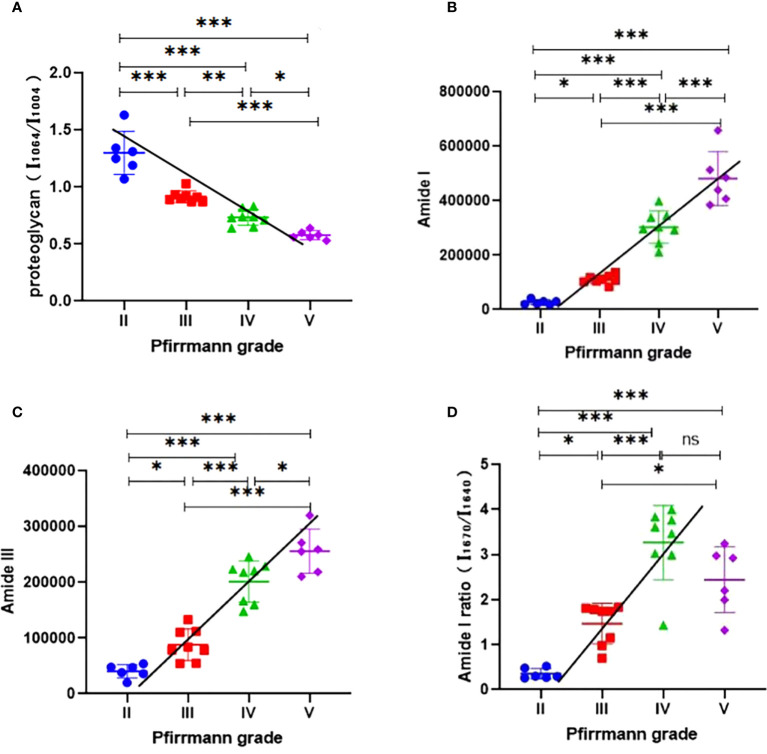
**(A)** Analysis of the relative proteoglycan content (*I*_1,064_/*I*_1,004_) in different Pfirrmann grades; the Spearman’s correlation analysis indicated that the relative proteoglycan content (*I*_1,064_/*I*_1,004_) decreased with the increase of Pfirrmann grades. **(B)** Analysis of the content of collagen (amide I) in different Pfirrmann grades; the Spearman’s correlation analysis showed that the content of collagen (Amide I) increased with the development of Pfirrmann grades. **(C)** Analysis of the content of collagen (amide III) in different Pfirrmann grades, the Spearman’s correlation analysis determined that the content of collagen (amide III) increased with the progression of the Pfirrmann grades. **(D)** Analysis of the intensity ratio of two peaks (*I*_1670_/*I*_1640_; amide I) in different Pfirrmann grades; the Spearman’s correlation analysis found that the intensity ratio of two peaks (*I*_1670_/*I*_1640_; Amide I) was significantly positively correlated with the Pfirrmann grade. ^∗^ *P* < 0.05; ^∗∗^ *P* < 0.01; ^∗∗∗^ *P* < 0.001; ns, no significance; *I*, relative intensity.

The relative proteoglycan content was found to be significantly reduced in Pfirrmann grades III, IV, and V compared with grade II (*P* < 0.001). According to Spearman’s correlation analysis, the relative proteoglycan content showed a dramatically negative correlation with the Pfirrmann grade (*ρ* = -0.9654, *P* < 0.001, **Figure 5A**).

As for the collagen content, the amide I group as well as the amide III group within it were analyzed separately. The amide I content was significantly higher in Pfirrmann grades III, IV, and V compared with grade II (*P* < 0.05, *P* < 0.001, and *P* < 0.001, respectively). Spearman’s correlation analysis indicated a highly positive correlation between amide I content and Pfirrmann grade (*ρ* = 0.9629, *P* < 0.001, **Figure 5B**). The content of amide III in Pfirrmann grades III, IV, and V was significantly increased compared with that in grade II (*P* < 0.05, *P* < 0.001, and *P* < 0.001, respectively). Spearman’s correlation analysis demonstrated that the content of amide III was also significantly positively correlated with the Pfirrmann grade (*ρ* = 0.9312, *P* < 0.001, **Figure 5C**).

### Analysis of collagen structure in the samples

We found that the intensity ratio of the two peaks (*I*_1,670_/*I*_1,640_; amide I) in grades III, IV, and V was significantly higher compared with that in grade II (*P* < 0.05, *P* < 0.001, and *P* < 0.001, respectively), and the intensity ratio of the two peaks (*I*_1,670_/*I*_1,640_; amide I) had shown a significantly positive correlation with the Pfirrmann grade by Spearman’s correlation analysis (*ρ* = 0.8598, *P* < 0.001, **Figure 5D**).

## Discussion

To the extent of our knowledge, the present study was the first to characterize the composition and structure of the NP in patients with CDH by Raman spectroscopy, an emerging optical instrument. The Pfirrmann grade was proposed as an early semi-quantitative clinical assessment method to classify IVDD by disc structure, MRI signal intensity, height of disc, and the distinction between NP and AF. The Miyazaki grade (25) was introduced as a subsequent grading system for cervical degeneration since its grading criteria were similar to the Pfirrmann grading, and in view of our team’s previous study on lumbar disc degeneration according to the Pfirrmann grade, we choose this grading criteria as a standard reference. Due to the shortcoming of imprecise quantitative analysis of Pfirrmann, the IVDD grading results were assessed by *κ* statistics analysis (*κ*1 = 0.620 and *κ*2 = 0.614); therefore, the grading result is indeed credible. With MRI as one of the common clinical diagnostic techniques for intervertebral disc degeneration, it is more likely to determine disc degeneration in terms of some coefficients such as apparent diffusion coefficient and diffusion-weighted imaging. A previous study has also shown that segmental quantitative T2 grading was able to assess the degree of IVDD (26). However, the connection between these imaging parameters and extracellular matrix changes in IVDD has not been exactly elucidated. We analyzed the correlations between NP T2 signal intensity value and proteoglycan relative content using the bivariate correlation analysis and found that there was a significant correlation between them (*P* = 0.912 and *P* < 0.01) at the 0.01 level (two-tailed). The property of proteoglycans to maintain disc hydration further explains the changes in the MRI T2-weighted signal. Regrettably, it is still not yet possible to characterize the content and structure of the macromolecules within NP through MRI.

Raman spectroscopy is used to determine the composition of a substance by collecting the light scattered from the irradiated substance compared with the characteristic peaks and indirectly reflects the substance content by detecting the intensity of these peaks (27). Proteoglycans, as the main component of NP, form the network of polysaccharide polymers by binding multiple glycosaminoglycan chains in order to maintain intervertebral disc infiltration and hydration properties (28). A pilot study demonstrated that higher AF/NP glycosaminoglycan ratios tended to associate with higher MRI T2 grades (29). Moreover, we observed that the relative content of proteoglycans (*I*_1,064_/*I*_1,004_) measured by Raman spectroscopy was comparable with the NP T2 signal intensity value. It is worthwhile to mention that both studies illustrated the decrease in proteoglycan content. The previous study found that proteoglycan content was negatively correlated with Pfirrmann grade by light microscopy examination (30). In terms of experimental protocol, this study applied more advanced microscopic techniques to carry out experiments based on changing the animal model to the isolated NP. This is somewhat consistent with the data that we have tested; the relative content of proteoglycans in NP was significantly correlated with Pfirrmann grade. In our previous study, Raman spectroscopy of proteoglycans (*I*_1,064_/*I*_1,004_) in the NP of lumbar disc herniation revealed a significant correlation between Pfirrmann grades IV, V, and III, while no significant differences were found between grades IV and V (31). This is somewhat inconsistent with the results of this study. We consider this because of the uniquely anatomical characteristic of the cervical vertebrae; the cervical intervertebral discs have more probability to degenerate. In the present study, we demonstrated that the proteoglycan content decreased during the progression of the Pfirrmann grade. This also provides some statistical support for the MRI T2-weighted signal intensity change results in our study.

Another significant component within the NP, type II collagen, provides a meshwork for the proteoglycans to attach to while enabling the proteoglycans to be wound more tightly to cope with the corresponding compression and tension forces (32). In the present study, we assayed the major amide moieties of collagen to characterize itself by Raman peak position, respectively. The Raman spectroscopy analysis indicated that the content of amide I and III in grades III, IV, and V was significantly higher compared with that in grade II. As for the Spearman’s correlation coefficient, we found that both amide I and amide III contents had a significantly positive correlation with Pfirrmann grade. Our team found similar changes in the NP with lumbar disc herniation; the content of amide I in grades IV and V was higher than in grade III. With regards to amide III, there was no correlation between Pfirrmann grades III and V (31). The interpretation that we think is such that the composition of the disc also varies with level in the spine, with the collagen content of the NP being highest in the cervical discs and lowest in the lumbar discs (33), so the amide III content may be slightly different. We also hypothesized that this may be due to a decrease in the proteoglycan content of the NP and a relative compensatory proliferation of collagen in response to this degenerative condition. In addition, it is also not excluded that the structure of the NP may be further disturbed, and there is the possibility of the outer fibrous ring to break through to the inner layer. The structural disorders of the NP may further lead to the intervertebral disc being dehydrated, deflated, and hardened, which may make the process of herniation more susceptible to happen.

Raman spectroscopy could not only detect changes in NP collagen content but also characterize its secondary structure including α-helices, β-sheets, and random coils (34, 35). Amide I and III groups are the main expressive groups on the Raman spectrum; therefore, the secondary structures of both are usually characterized separately. A previous study indicated that the intensity ratio of the two peaks (*I*_1245_/*I*_1270_) provides information about the relative content of random *versus* ordered coils in the protein structure and the progression of cartilage dysregulation (36). In our study, we compared the relative contents of disordered coils *vs*. ordered coils to further determine the degree of disorder in the secondary structure of collagen. It was found that the intensity ratio of the two peaks (*I*_1,670_/*I*_1,640_; amide I) was significantly positively correlated with the Pfirrmann grade. Compared with the previous study that we have conducted (31), the present study results include additional Pfirrmann grade II samples and show the comparable results with before. The increase in disordered collagen components further aggravated the degree of structural disorder in NP. These results characterize the secondary structure of disordered collagen by Raman spectroscopy and further have the potential to elucidate the microstructural mechanism of IVDD.

It should be emphasized that this study has several limitations. First, the number of samples that we included was relatively limited, and there was still a lack of some degree of classical histological basis. In future studies, a larger sample composition and experiments like IHC will be applied to further investigate the mechanism of IVDD. Second, the irregularities on the surface of biological materials can significantly affect the peak intensity and background, making the high background expression inherent to the Raman spectroscopy detection process unavoidable. Furthermore, the study was designed for *in vitro* NP samples; thus, the comparative effectiveness of Raman spectroscopy in detecting the composition and structure of macromolecules still needs to be elucidated by *in vivo* studies.

In summary, our results demonstrated that content of macromolecules like proteoglycan and collagen during IVDD and, most importantly, provided a certain elaboration of this process at the microscopic level. The higher relative intensity of the two peak ratios (*I*_1,670_/*I*_1,640_; amide I) detected by Raman spectroscopy may have the potential to become an early effective indicator for the detection of IVDD.

## Data availability statement

The raw data supporting the conclusions of this article will be made available by the authors without undue reservation.

## Ethics statement

This study was reviewed and approved by the Ethics Committee of Tianjin First Central Hospital. The patients/participants provided their written informed consent to participate in this study.

## Author contributions

TZ and ZL conceptualized and designed the study. ZW, XW, TX Z, and EG contributed to the collection and preparation of the samples. TX, ZL, and ZW performed the Raman spectroscopy measurement. ZW and HZ contributed to the acquisition of data. ZW and XW performed the data analysis. ZG and QZ supervised and directed the project. All authors contributed to the article and approved the submitted version.

## Funding

This work was supported by a research grant from the Science and Technology Project of the Tianjin Municipal Health Commission (project number MS20020).

## Acknowledgments

Thanks to all the teachers and students at the Department of Orthopedics, Tianjin First Central Hospital and the School of Materials Engineering Science and Engineering, Tianjin University.

## Conflict of interest

The authors declare that the research was conducted in the absence of any commercial or financial relationships that could be construed as a potential conflict of interest.

## Publisher’s note

All claims expressed in this article are solely those of the authors and do not necessarily represent those of their affiliated organizations, or those of the publisher, the editors and the reviewers. Any product that may be evaluated in this article, or claim that may be made by its manufacturer, is not guaranteed or endorsed by the publisher.
